# Preoperative Cholinergic Signatures Drive Segregated Brain Architecture in Postoperative Delirium

**DOI:** 10.21203/rs.3.rs-7881643/v1

**Published:** 2025-11-23

**Authors:** Natasha Taylor, Brandon Munn, Klevest Gjini, Isabella Orlando, Benjamin Moran, Jennifer Taylor, Jordan Wehrmann, Matthew Banks, Veena Nair, Robert Pearce, David Kunkel, Vivek Prabhakaran, Richard Lennertz, James Shine, Robert Sanders

**Affiliations:** The University of Sydney; University of Sydney; University of Wisconsin; University of Sydney; Department of Anaesthesia, Gosford Hospital; The University of Sydney; The University of Sydney; University of Wisconsin School of Medicine and Public Healtth; UW MADISON; University of Wisconsin; UW; University of Wisconsin - Madison; University of Sydney; University of Sydney

**Keywords:** Delirium, Resting-state fMRI, Cholinergic, Segregation, Gene expression, Ascending Arousal Systems

## Abstract

Delirium affects up to 50% of hospitalised, older patients and is linked to increased risk of death and long-term cognitive decline. Age-related changes in the ascending arousal system (AAS), including cholinergic and noradrenergic nuclei, may contribute to delirium vulnerability. Static and dynamic functional connectivity, across cortical and subcortical regions, was extracted from preoperative resting-state fMRI from 120 older adults (aged > 65 years old). Participants who developed delirium showed more segregated brain networks, cholinergic hyperconnectivity and noradrenergic hypoconnectivity. Dynamic patterns from these systems separated groups in low-dimensional space, suggesting altered temporal network dynamics. Normative maps of cholinergic gene expression density were associated with increased network segregation. These results suggest that aging-related AAS alterations—particularly compensatory cholinergic overactivity—may drive network changes that increase delirium risk. This work provides new insights into the neural mechanisms linking aging, arousal system dysfunction, and brain network disruption in delirium, mandating re-appraisal of leading delirium theories.

## Introduction

Delirium is an acute state of cognitive impairment, attention, and/or arousal that has significant impact on overall long-term cognitive outcomes and is associated with an increased risk of dementia^1^. It disproportionality impacts the aging population with up to 56% of elderly hospitalized patients affected^2^ and advanced aging (> 65yrs) being a significant risk-factor due to neuroinflammatory changes^3^.

Critically it is associated with increased morbidity, mortality, and costs^4^. Amongst the prevailing theories, one suggests that delirium can be described as a state of ‘cognitive disintegration’ due to a reduction or dysfunctional connectivity between specific cortical networks that are necessary for maintaining normal cognitive function^5^. The theory predicts that delirium results when the network connectivity disintegrates below a critical threshold. While this theory is supported by recent EEG studies, showing a reduction in feedback cortical connectivity^6^ and resting state functional connectivity (FC) in delirium^7–10^, data linking connectivity to vulnerability are less forthcoming. Several studies have revealed that global and regional cortical atrophy, white-matter hyperintensities and structural based differences may predict postoperative delirium^7,11–13^. These studies revealed that neuropathological changes in postoperative delirium are detectable with standard functional imaging techniques; however they have not established directly whether there are pre-existing dynamic and static FC signatures that are indicative of vulnerability to delirium, that may provide important insights into disturbed neural dynamics.

Aligning with the cognitive disintegration theory is a graph theoretical measure popularised in network neuroscience; participation coefficient. Participation coefficient quantifies how evenly distributed a region’s connections are within its own network/subnetwork compared to its connections to regions outside of their network^14^. As such, this is used to define whether brain network topology is either integrated (strong connections across brain networks) or segregated. Network topology has been shown to be disrupted across several neurodegenerative diseases of aging, specifically in Alzheimer’s disease^15^ (AD) and Dementia with Lewy Bodies^16^ (DLB). This highlights that in other neurodegenerative conditions, that delirium populations are more at risk of developing^1^, there is an overlap in underlying network topological changes.

Patterns of brain wide integration and segregation have been associated with underlying neuromodulatory systems, the noradrenergic and cholinergic neuromodulators. Previous work has evidenced that activity within the noradrenergic locus coeruleus (LC) can imbue the system towards a more integrated network topology^17,18^ and is critical in exploitation/exploration cognitive processes^19^. Whereas, the cholinergic system has been thought to facilitate a more segregated network topology due to its more targeted projection patterns across the cortex, which is critical for selective attentional control^20,21^. This highlights a preoperative vulnerability in the ascending arousal systems that relates to cognitive disintegration theory.

The noradrenergic and cholinergic systems are critical for normal cognitive function and; they are sites of substantial pathology in neurodegenerative diseases^22^. Specifically, the noradrenergic locus coeruleus undergoes early pathological degeneration across neurodegenerative disease^23–25^. The integrity of the locus coeruleus has been extensively linked to decline in memory performance in ageing and in Alzheimer’s disease^26–29^. Given noradrenaline’s critical role in facilitating selective attention^30,31^; preoperative vulnerability within the locus coeruleus could underly the neuropathological insult prior to surgery. In addition to the loss of the noradrenergic system integrity, there is substantial evidence of cholinergic associated cognitive decline in both Lewy Body Dementia^32–34^ and Alzheimer’s disease^35–38^. Cholinergic pathology precedes degeneration in other associated brain regions and has been shown to predict future memory impairment in non-symptomatic aging individuals^39,40^. This results in dysfunction in learning, memory, sensory and attentional processing^41^ as the cholinergic system is critical in modulating feedforward processing within the cortex^42–44^. Recent literature has also linked non-linear interactions of the cholinergic system: increases in cholinergic presynaptic VAChT levels colocalized with tau is predictive of cognitive resilience in older adults at risk of AD^45^. Consistent with this, we have recently observed exaggerated feedforward processing in individuals who are vulnerable to delirium^46^ and in patients with tau disease on PET imaging^47^. As such, dysfunctional noradrenergic and cholinergic neuromodulation could underly vulnerability to delirium, with early degeneration of the locus coeruleus and subsequent non-linear compensatory hyperactivity of the cholinergic system. Given the cognitive disintegration theory, we hypothesise that preoperative participants that go onto develop delirium will have dysfunctional network topology, with a greater degree of segregation (disintegration) related to disturbed function of the ascending arousal system.

## Results

A total of 296 participants were recruited for this study. 153 participants did not undergo preoperative MRI scans due to refusal of consent or ineligibility. Delirium occurred in 32.4% of scanned participants and 37.0% of non-scanned participants. After performing pre-processing and denoising of the resting state fMRI data, a total of 120 participants (see methods for exclusion) were analysed. The delirious scanned participants were matched to non-delirious participants across most characteristics (see supplementary materials Table 1), except for operative severity (National Surgical Quality Improvement Program risk of Surgical Complications or Death) and a marker of peak delirium severity, overall peak Delirium Rating Scale-98 (DRS). These analyses compare *before surgery* (preoperative) between participants who later developed postoperative delirium (termed delirious) and who did not develop postoperative delirium (termed non-delirious), scanning was conducted when no patient was actively delirious.

**Figure 1:**
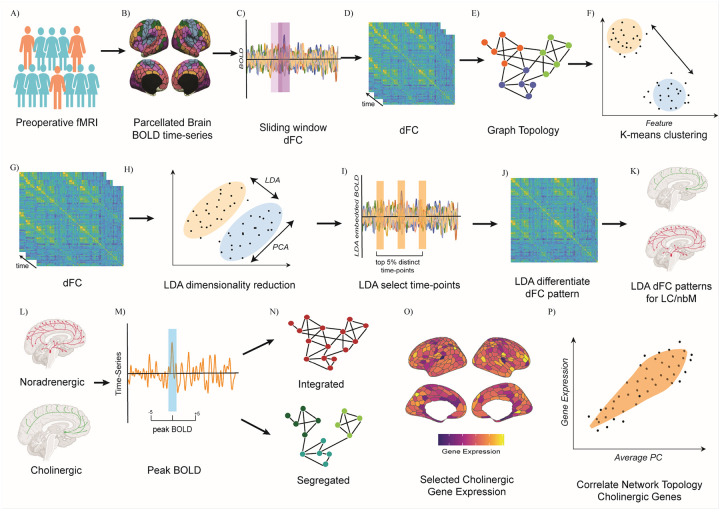
Illustration of different methods for the following analyses. A) Preoperative participants were selected for resting-state functional MRI. B) Preprocessed and denoised fMRI BOLD signal was extracted across 400 cortical regions using the Schaefer parcellation and 2 ascending arousal nuclei (locus coeruleus and nucleus basalis of Meynert). C) Sliding-window approach dynamic functional connectivity using multiplication temporal derivatives. D/G) Dynamic functional connectivity produces correlation matrices across time. E) Graph Topology, using the Louvain community detection algorithm to calculate participation coefficient. F) K-Means clustering was performed to differentiate states of integration or segregation. H) Linear discriminant Analysis was used to maximally separate spatio-temporal patterns of dFC across the delirious and non-delirious. I) Top 5% of separating time-points were selected from the LDA embedded time-series. J) Average of the top-selected time points across the dFC for the delirium and non-delirious groups. K) Coupling patterns of the LDA differentiate dFC patterns for the LC and nbM. L) Selecting BOLD time-series from the LC and nbM. M) Peak BOLD time-series for the LC and nbM were calculated based of 2 standard deviations. N) Cross-correlation was performed with the peaks in BOLD time-series from the LC and nbM versus changes in the participation coefficient overtime. O) PCA reduced gene expression maps of the cholinergic system were preselected from the Human Allen Brain Atlas. F) Correlation between the cholinergic gene expression and the average participation coefficient value was calculated.

To disentangle the contributions of the ascending arousal system to segregated network topology in development of postoperative delirium; time-series were extracted across 400 cortical regions^48^, the pontine locus coeruleus (cortical source of noradrenaline)^49^ and basal forebrain nucleus basalis of Meynert (source of acetylcholine)^50^. Pearson’s correlation was calculated to estimate static (FC) and dynamic functional connectivity (dFC) was calculated using the multiplication of temporal derivatives^51^. We calculated graph theoretical derived segregated network topology using participation coefficient and determined distinct spatio-temporal dFC patterns using linear discriminant analysis in those delirious compared to non-delirious. Lastly, we related cholinergic gene expression density maps^52^ to regional segregation across the brain (Fig. 1 – graphical methods).

### Segregated Network Topology in Postoperative Delirium

**Figure 2:**
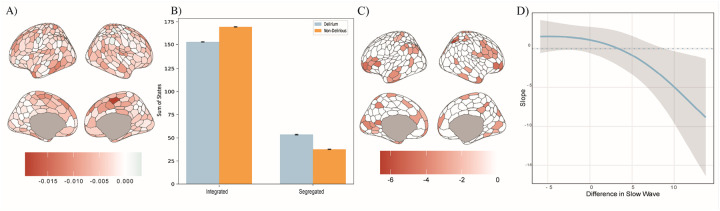
Significant difference in participation coefficient values between delirium and non-delirious. A) Significant difference in average participation coefficient values, delirium – non-delirious (p<0.05, 1000 iterations). B) K-means clustering of the entire participant coefficient across all regions over time, clustered into integrated and segregated states and grouped into delirium (light blue) and non-delirious (yellow). C) Logistic regression of the significant beta coefficients for predicting delirium outcome and interaction between participation coefficient and slow-wave EEG activity. D) Exemplar fitted curve plot of logistic regression of predicting delirium outcome and interaction between regional average participation coefficient (Default B network) and difference in slow-wave EEG activity.

We explored time-varying network topology by calculating the dynamic functional connectivity (dFC) and used participation coefficient (PC)^14,53^ to quantify the distribution of connections across different network modules. We determined whether there were significant differences in integration (larger PC) versus segregation for the delirious compared to non-delirious participants (1000 iterations of permuted p-value). We found that on average across the time-series, the delirium participants had a relatively decreased PC score compared to the non-delirious participants (mean pc delirium = 0.39 ± 0.05, mean pc non-delirious = 0.41 ± 0.04; Fig. 2A), suggesting that the delirium population had more segregated network topology. Specifically, this was driven by a greater segregation across the salience attention and default mode networks. To further interrogate this, we ran a k-means clustering^53^ across the entire time-series for all cortical regions and found that the delirium population was more frequently in a segregated state compared to the non-delirious population (Fig. 2B). In addition, to determine whether the preoperative PC was an important biological predictor of delirium, we adjusted for the main biological neural correlate *during* delirium – postoperative EEG slow wave activity^7,54^. This adjustment is important to confirm a robust relationship between segregation and delirium. We verified that the preoperative PC value interacted with difference in pre versus postoperative EEG slow wave activity (change Oz-delta power) in their association with postoperative delirium using logistic regression (Fig. 2C, evidenced with fitted curved plot in Fig. 2D). These data demonstrate a negative interaction between PC and EEG slow wave activity: as PC falls and slow wave activity increases, the probability of delirium increases. Furthermore, the adjustment for slow wave activity confirms that PC predisposes to delirium independent of the change in brain electrophysiology that occurs during delirium.

### Noradrenergic and Cholinergic Dysfunctional Static Connectivity

**Figure 3:**
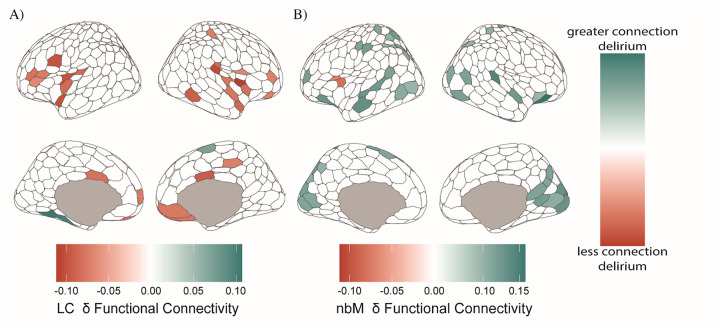
Significant difference in functional connectivity in noradrenergic and cholinergic systems. A) Significant difference in functional connectivity from locus coeruleus, delirium – non-delirious (p<0.05, 1000 permutation test). B) Significant difference in functional connectivity from PPN. C) Significant difference in functional connectivity from nbM to whole cortex.

We found significant hypoconnectivity between the LC and the pre-frontal regions and anterior cingulate of the cortex for the delirium population compared to non-delirious (Fig. 3A). We found a significant hyperconnectivity between the nbM and the posterior and superior temporal cortical regions in the delirium compared to non-delirious participants (Fig. 3B). In addition, the simple logistic regression results for predicting delirium outcome based on ascending arousal nuclei connectivity found that hypoconnectivity from the LC to salience attentional (p = 0.041), and control networks (p = 0.034) were associated with delirium. Decreases in connectivity from nbM to regions within peripheral visual (p = 0.017) and default network (p = 0.045) and increased connectivity from nbM to central visual network (p = 0.016) were also associated with delirium (Supplementary Materials, Fig.S3). In addition, inspired by a recent report^45^, in a post hoc analysis we show a positive linear relationship between stronger nBM FC and pTau (blood biomarker), (Supplementary Materials, Fig. S9); however the regional differences were not significant after FDR correction.

### Dynamic Functional Connectivity Ascending Arousal Patterns

**Figure 4:**
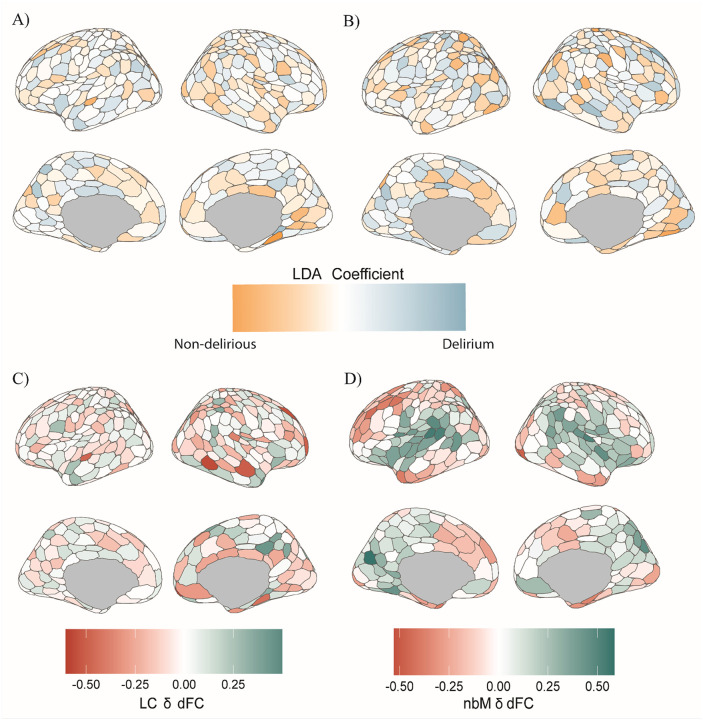
Linear Discriminant Analysis of dynamic functional connectivity of ascending arousal nuclei that differentiate delirium and non-delirious preoperatively. A-B) Linear Discriminant Analysis coefficients that determine which regions are distinct in differentiating the delirium and non-delirious participants ran on the time-varying components of the dynamic functional connectivity for LC (A), and nbM (B) (blue indicates delirium related, orange indicates non-delirious). C-D) LDA defined time-points that maximally differentiate the delirium and non-delirious participants (through LDA embedding of the dFC), taking an average of these time-points for the dFC and calculating the difference between the delirium and non-delirious groups across ascending arousal nuclei from LC (C) and nbM (D) to entire cortex.

We next explored time-varying features of brain connectivity. We used eigen-mode linear discriminant analysis (LDA) to determine specific spatio-temporal patterns that are unique to the delirium and non-delirious populations across the LC dFC and nbM dFC (Fig. 4A–B). The dFC matrices were concatenated across subjects and time, scaled and balanced-class priors for LDA was used to identify features contributing to maximal separation between the groups. Using the highest-order magnitude (top 5%, based LDA coefficients) time-points across the dFC that separate the two groups, the LDA-embedded average dFC were compared for noradrenergic and cholinergic connectivity between delirious and non-delirious groups. We found greater anti-coupling between the LC and frontal regions in the delirium population compared to non-delirious (Fig. 4C). The nbM had greater coupling across posterior-temporal regions and distinct regions within the default mode network (DMN) in the delirium group compared to non-delirious, and there was anti-coupling across regions within the salience attentional and somato-motor networks (Fig. 4D). These heterogenous patterns were mirrored when looking at average dFC differences between the groups (Supplementary Materials, Fig. S4).

**Figure 5:**
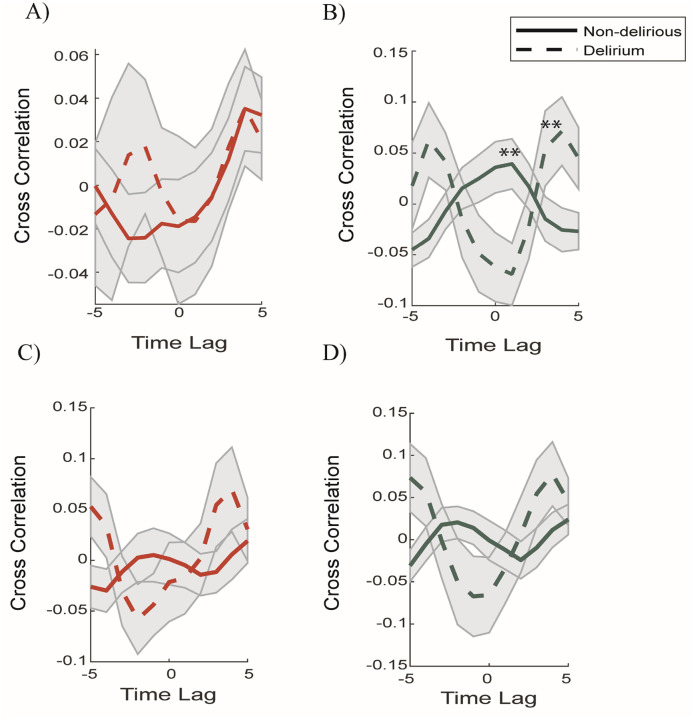
Peak activity in BOLD time-series for ascending arousal nuclei associated with greater integration & segregation. Peak time-series cross-correlated to global participation coefficient across time separated into delirium and non-delirious, for LC (A), nbM (B). Peak difference in time-series cross-correlated with global participation coefficient separated into delirium and non-delirious for C) LC – nbM (D) nbM – LC. ** significant difference between two groups for time-lags for nbM peak p=0.04 (Lag 0), p=0.0150 (Lag +1), p=0.02 (Lag +4), p=0.049 (Lag +5).

We were interested in determining whether there are time-dependent relationships between the global brain integration/segregation compared to ascending arousal BOLD dynamics. Leveraging an approach from Munn et al., (2021) derived from younger individuals^55^, we calculated the cross-correlation between the phasic peaks (defined by 2-standard deviations) of the BOLD signal for the LC and nbM against the average (global) participation coefficients for each subject. Similar to Munn et al., (2021), we found a mirroring pattern, wherein post-phasic LC activity was positively correlated to greater integration (Fig. 5A) and post-cholinergic activity was related to more segregation (Fig. 5B–D). However, the cross-correlation between nbM and global PC occurred in bursts: there was greater segregation for the delirium population for time-lag 0 (p = 0.04) and time-lag + 1 (p = 0.015) compared to non-delirious (Fig. 5B), but the relationship became flipped at greater time-lags. This finding is consistent with a hyperactive cholinergic response in the delirium population driving greater segregation.

### Relationship between Segregation and Gene Expression Receptors

**Figure 6:**
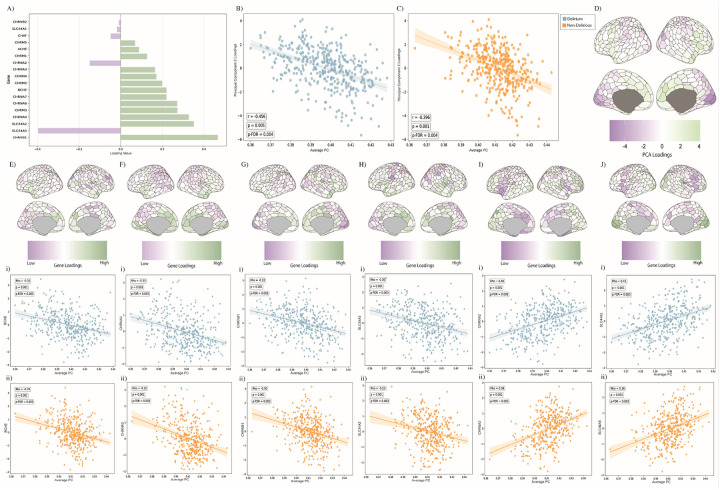
Relationship between Human Allen Brain Atlas derived Cholinergic Gene Expression and Segregated Network Topology. A) The principal component coefficient loadings for the pre-selected cholinergic genes in the second principal component. B) The Pearson correlation between the average participation coefficient for each 400 cortical ROIs for the delirium population versus second principal component loadings of pre-selected gene expression, p_spin_ = 0.001 (1000 iterations) and FDR corrected p=0.004. C) The Pearson correlation between the average participation coefficient for each 400 cortical ROIs for the non-delirious population versus second principal component loadings of pre-selected gene expression, p_spin_ = 0.001 (1000 iterations) and FDR corrected p=0.00. D) Preselected gene expression second principal component loadings across the Schaefer cortical parcellation. E-J) Visualising the selected gene expression loadings on the Schaefer cortical parcellation. i)the Pearson correlation between average participation coefficient for all participants compared to selected (E-J) gene expression (p_spin_ <0.05, FDR correction). E) BCHE = butyrylcholinesterase. F) CHRNA3 = nAChR 3 subunit. G) CHRNB1 = nAChR ß subunit. H) SLC44A2 = choline transporter-like protein 2. I) CHRNA2 = nAChR 2 subunit. J) SLC44A5 = choline transporter-like protein 5.

Lastly, we explored whether regional segregation related to gene expression derived from the Allen Human Brain Atlas^52,56,57^. We selected genes relating cholinergic receptor expression, neurotransmission and clearance enzymes and performed principal component analysis to reduce the dimensionality of the number of genes (see Methods). Then, we correlated the principal component loadings (across 8 principal components, Supplementary Materials, Fig. S6) to the regional average PC across the delirious and non-delirious participants combined (and distinct; Supplementary Materials, Fig. S7). The top performing linear relationship was ranked and validated based upon largest effect size (*r* = 0.3, p = medium effect) and greatest absolute correlation coefficient (see Methods). We found a strong negative relationship between the second principal component of the cholinergic related gene expression correlated with the average PC across both the delirium (rho=−0.456, p_spin_ = 0.001, p-FDR = 0.004) and non-delirious (rho=−0.396), p_spin_ = 0.001, p-FDR = 0.004) participations (Fig. 6B–C). Importantly, the second principal component embeds nicotinic receptors (nAChRs) and cholinergic neurotransmission gene-related expression (Fig. 6A) with higher gene loadings across frontal cortical regions (Fig. 6D). In addition, when observing the individual cholinergic gene expression related to the average PC; several genes that contribute to the loading in the second principal component are significantly negatively related to PC across both groups (Fig. 6E – H), across nicotinic receptors (Fig. 6-F–G) and butyrylcholinesterase (Fig. 6E). Interestingly, two distinctive genes that have negative loadings in the second principal component show a positive relationship with average PC (Fig. 6I – Ji) and the gene expression is inverted with low expression across frontal and high loadings across posterior cortex compared to all the other expression maps (Fig. 6I #x2013; J). This is additional evidence that there is a mechanistic relationship between regional cholinergic gene expression and segregated network topology. We also examined the relationship between cholinergic gene expression and nbM functional connectivity, however none of these results passed FDR correction (Supplementary Materials, Fig. S8).

## Discussion

In this study, we found signatures of disrupted ascending arousal modulation in preoperative resting-state scans of participations who developed postoperative delirium. Preoperatively, delirious participants had more segregated network topology and evidence of disrupted ascending arousal modulation compared with non-delirious participants, with decreased connectivity between the LC and cortex and hyperconnectivity between cholinergic nbM and the posterior regions of the cortex.

The relative reduction in the preoperative participation coefficient across the entire cortex for individuals with postoperative delirium was explored through a data-driven approach to categorise the segregated and integrated states across the entire time-series for all cortical brain regions. We found that the participants who were vulnerable to postoperative delirium spent more time in a segregated state compared to non-delirious participants (Fig. 2B). Furthermore, we established a relationship between the interaction with slow-wave activity (a key biological correlate of the precipitant of postoperative delirium^7^) and segregated brain regions in predicting postoperative delirium outcomes (Fig. 2C). This establishes a robust relationship between delirium and the segregated network topology of the population. Segregated network topology has been evidenced across several other neurodegenerative conditions, including Dementia with Lewy Bodies^16^, Alzheimer’s disease.^58^ Within the postoperative population there is evidence of segregated networks in EEG analysis.^59^

Previous large data analysis of familial Alzheimer’s disease (ADNI study) has found that greater segregation of higher-order cognitive networks is critical in mediating cognitive resilience despite neuropathology^60^. This finding parallels our findings as there were no preoperative differences in cognition between those who became delirious compared to those that did not. Hence, greater segregation in the delirium population, highlights a potential marker of compensatory cholinergic activity. These findings support the cognitive disintegration theory^5^ and extend it to include pre-existing ascending arousal changes prior to surgery.

We further interrogated the relationship between the ascending arousal nuclei activity and the network topology by performing a time-lagged cross-correlation analysis between peaks in BOLD activity of the ascending arousal system nuclei and the global brain segregation. Our results replicated previous findings^20,55^ in which increased integration is positively related to peaks in LC activity (Fig. 5A), across both delirium and non-delirium participants. However, we found that the cholinergic nuclei exhibited an inverse relationship, suggesting that post phasic peaks in cholinergic activity are related to greater segregation; with a significant disproportionate relationship between greater segregation post-phasic nbM activity in the delirium population (Fig. 5C), this result provides a potential explanation for the overall segregated topology in the delirium participants (Fig. 2A). It also supports the theory that the cholinergic system may drive a more segregated network topology through facilitation of normalization^61^ by emphasizing patterns of neuronal activity through modulation of inhibition and an increase in the signal-to-noise ratio in these targeted regions^61,62^. Given the hyperconnectivity of the nbM to the cortex in the delirium population, we speculate that vulnerability to delirium relates to mechanistic compensatory changes in the cholinergic system that drive greater segregation.

We extended this analysis to determine whether there is a relationship between the regional participation coefficient and gene-expression for cholinergic receptors and clearance enzymes. We found a negative relationship across multiple nicotinic sub-unit receptor genes (Fig. 6F–G) indicating that the more segregated regions have specific cholinergic receptor-related gene expression and may therefore be more responsive to changes in cholinergic modulation^63^. Importantly, nAChRs have been related to executive control and higher-order cognition, mirroring by the pattern of gene expression loadings across frontal-cortical regions (Fig. 6D).^63,64^ There was observational overlap between greater segregation across the frontal-cortical regions (Fig. 2A) and the heterogeneity of distinct cholinergic gene expression across the frontal-cortical regions. Also, there was a strong negative correlation between butyrylcholinestrase (BCHE) gene expression and segregation in the delirium population (rho= −0.32, *p*_*spin*_= 0.001, FDR = 0.003). Previously, higher levels butyrylcholinestrase activity (in blood) have been associated with delirium^65^, indicating a potential relationship with butyrylcholinestrase activity and associated vulnerability in these regions. In addition to these findings, we also established a weakly positive relationship (rho = 0.157, p_spin_=0.003, FDR p = 0.008) between hyperconnectivity of the nbM and the first principal component of cholinergic gene expression, which proportionally represent muscarinic receptors M1 and M3 (Supplementary Materials, Fig. S6). These findings support the anatomical disparate projection patterns of the cholinergic system^66^ – specifically in relation to how the cholinergic system is driving segregated patterns; it may be facilitating greater segregation specifically in relation to regions that have higher-levels of nicotinic gene-expression.

We also found that the delirium population had heterogenous time-varying coupling patterns between the LC and nbM. We found differences in the functional connectivity of the noradrenergic system, with a relative decrease in connectivity for the delirium population preoperatively and anti-coupling in the dFC. These results further suggests that there is imbalanced ascending arousal function in the preoperative delirium population, as evidenced by dynamic features of the functional connectivity. The nbM appears to have more coupling across posterior cortical regions in the delirium participants compared to the non-delirious participants (Fig. 4B/D). This pattern is consistent across different functional connectivity profiles, including in static functional connectivity (Fig. 3B). The occipital/posterior related hyperconnectivity differences with the nbM could relate to an underlying vulnerability across these regions, as we see changes in slow-wave activity (from EEG) propagating across these posterior cortical regions.^7^ Our findings also parallel previous work that has shown potential compensatory hyperconnectivity in the preoperative at-risk delirium populations and across early AD pathology^67,68^. Additionally, microstructural white-matter changes within the basal forebrain (containing the nbM) predisposes individuals to postoperative delirium independent of cognitive function^69^. This suggests that perhaps in conjunction with functional related changes, there are axonal and structural based changes that could be related to mechanistic changes in neuronal vulnerability prior to the neuroinflammatory insult that occurs during surgery.

Taken together, these findings lead us to speculate that the cholinergic system predisposes to selective vulnerability in part due to early presynaptic adaptations aimed at maintaining plasticity and network function in the face of other neuronal pathologies^68,70,71^. Basal forebrain cholinergic synapses show an adaptive response to early tau pathology—potentially a compensatory mechanism—whereby presynaptic VAChT (Vesicular Acetylcholine Transporter) is upregulated in at-risk, cognitively resilient older adults, particularly when colocalized with tau rather than amyloid^45^. This adaptive response appears to mitigate tau-induced synaptotoxicity in cognitively resilient individuals^45^. Along these lines, in an preliminary exploratory analysis we showed a positive linear relationship between pTau (within the blood) and increased cholinergic connectivity^72^ (Supplementary Materials, Fig. S9) which could suggest a relationship with underlying cholinergic driven synaptic modulation and neuropathological accumulation; as our participants are cognitively healthy (see Supplementary Table S1). Given that acute inflammation is known to drive central cholinergic suppression, our findings suggest that those predisposed to delirium can be conceptualised as more susceptible to the neuroinflammatory insult during surgery and unable to compensate for loss of cholinergic tone in the brain. While this reinvigorates the decades old cholinergic hypotheses for the mechanisms of delirium^73^, our data suggests that the nicotinic receptor family should be specifically considered as well as protocols that allow for dose-dependent modification.

A limitation of this study is the potential selection bias between scanned and non-scanned participants. Individuals who were not scanned are likely to have undergone more complex surgeries and/or presented with more severe comorbidities, which may reduce the generalisability of our findings. All participants were offered scanning, unless contraindicated (e.g. by surgical implants), and so the difference in the population primarily relates to participant choice. Nonetheless this does limit the broader generalisability of our work. Additionally, there are limitations in relation to other unknown confounders that cannot be fully excluded. Importantly, cognitive measures previously associated with delirium risk showed no significant differences between delirious and non-delirious participants (Table 1), nor between scanned and non-scanned individuals (Supplementary Table 1). This enhances confidence that any observed resting-state functional connectivity differences reflect preoperative neural vulnerabilities, rather than cognitive disparities. Our findings diverge from prior work suggesting that known delirium risk factors (e.g., age, cognitive impairment, depression) are not associated with delirium-related network changes in fMRI.^9^ However, that prior study employed distinct methodological approaches, that likely account for the discrepancies in our findings. In addition, the use of the Human Allen Brain Atlas cholinergic gene expression does not represent individual variations in gene expression, which is a limiting factor of this analysis. Future research could aim to incorporate multimodal neuroimaging, such as simultaneous PET-fMRI and structural imaging, to further elucidate potential neuropathological mechanisms underlying ascending arousal related differences in populations at risk for delirium.

## Conclusion

Given that postoperative delirium is associated with later-life cognitive decline^74^, and neuronal injury^75^, a postoperative delirium event might act as a further insult to an already vulnerable ascending arousal system. Further research needs to interrogate whether there are lasting disruptions to the ascending arousal system postoperatively. This would be critical in revealing the relationship between postoperative delirium and associated cognitive decline.

## Data availability

The data that support the findings of this study are available upon reasonable request from the corresponding author. The data are not publicly available due to clinically relevant data under ethics restrictions. All code for the analysis is available: https://github.com/NatashaLTaylor/Preoperative_fMRI_AscendingArousal

## Methods

### Clinical Data

All data were collected as part of the perioperative registered trial (2015–0374, NCT01980511 and NCT03124303) cohort study, Interventions for Postoperative Delirium: Biomarker-3 (IPOD-B3), approved by University of Wisconsin-Madison Institutional Review Board (2015–0374). 5519 All participants provided informed consent, and work was conducted in accordance with the Code of Ethics of World Medical Association (Declaration of Helsinki) for experiments involving humans. 5519 individuals were screened, and 296 patients were enrolled for participation in the perioperative cohort study. All participants were aged 65yrs or older and underwent major elective non-intracranial surgery, under general anaesthesia. 153 participants did not undergo preoperative MRI scans (due to refusal of consent or ineligibility). Delirium occurred in 32.35% of scanned participants and 37.04% of non-scanned participants. Exclusion criteria consist of any documented history of dementia or residing in nursing aged care facilities. Participants underwent pre-operative MRI scans across five sites. They were all assessed for up to 4 days post-operatively which included twice daily (between 05:00–10:00 and 16:00–20:00) delirium assessment using 3-minute Diagnostic Confusion Assessment Method (3D-CAM)^1^, or CAM for intensive care unit (CAM-ICU, if intubated), bloods were collected pre-operatively and, on each day, post-operatively for 4 days. After surgery, participants underwent delirium assessments twice daily using the 3D-Confusion Assessment Method (3D-CAM or CAM-ICU (dependent on whether patients were intubated or not) to define delirium. Additionally, in patients who were able to verbalise, delirium severity was assessed by delirium rating scale 98 and 3D-CAM-severity score. All assessors were trained in 3D-CAM^2^, CAM-ICU^3^ and delirium rating scale delirium assessments. Assessments were discussed in weekly meetings to review diagnoses and harmonise delirium scoring. Coma was assessed by Richmond Agitation/Sedation Scoring (RASS).^4^ All patients who scored for coma, later scored as delirious on formal testing. Daily blood sample collection was also conducted. Samples were analysed by NULISA^5^ but only pTau181 based on our prior work using this biomarker in delirium^6^ and a prior report implicating Tau associated changes with cholinergic signalling. EEG were collected preoperatively and on postoperative day 1. If patients were in a coma and could not be assessed for delirium the EEG was deferred until a delirium assessment was possible (see Tanabe et al, BJA 2020^7^). Three minutes of average re-referenced clean signals were obtained from high-density EEG data acquired at a 250 Hz sampling rate using a 256-channel system (Electrical Geodesics, Inc.). EEG data from a resting-state, awake, and eyes-closed condition were processed in EEGLAB^8^, a toolbox running in MATLAB (The MathWorks Inc., Natick, Massachusetts). Artifact detection and rejection was performed using independent component analysis (ICA). Delta band [1–3.5 Hz] power in dB units was calculated for electrode Oz using EEGLAB’s function ‘spectopo’ from the power spectral density (PSD) with Welch’s method (2-sec ‘hamming’ windows and with 50% overlap).

Cognition was assessed preoperatively using Montreal Cognitive Assessment (MoCA) Trail Making Test A/B (TMT-A & TMT-B), Boston Naming Test, Digit Symbol Substitution Test (DSST), Controlled Oral Word Association Test (COWAT) and years of education. The following intraoperative assessments were collected as the American College of Surgeons’ National Quality Improvement Program for surgical risk of death (NSQIP-D), National Quality Improvement Program for surgical risk of serious complications (NSQIP-SC) and American Society of Anesthesiologists Physical Status Classification (ASA). From a total of 142 scanned participants, seven subjects were removed due to incomplete overall peak DRS scores, as surgery was cancelled (n=3), two patients died during or shortly after surgery, one patient withdrew, and one patient surgery was converted to an outpatient procedure. A subsequent twelve subjects were removed due to substantial denoising/pre-processing errors in fMRI, and incomplete fMRI scan protocols (no resting-state scans completed, n=3).

### Functional MRI acquisition and preprocessing

Participants were scanned across five different sites with the following General Electric MRI scanner types: group 0 was scanned on a 3T GE 750 (n=35), group 1 was scanned on a 1.5T Optima MR 450 wide bore (n=2), group 2 was scanned on 3T GE 750 & 3Tw Premier (n=4), group 3 was scanned on a 3T Signa PETMR (n=77), group 4 was scanned on a 3Tw Premier (n=1) and group 5 was unknown (n=1). A General Electric MRI scanner was used to acquire T1-weighted axial structural images at beginning of each scan using FSPGR BRAVO sequence; repetition time = 8.132ms; echo-time = 450ms, 256 × 256 matrix, 156 slices, flip-angle = 60°, and slice thickness = 1mm. Resting state fMRI scanning was done using T_2_-weighted echo-planar functional images, acquired in interleaved order with: repetition time = 2600ms; echo-time = 22ms; flip-angle = 60°; 40 axial slices covering the entire brain; interslice gap = 3.5 mm; field of view = 224mm; and the raw voxel size = 3.5 × 3.5 × 3.5mm. Pre-processing of images was performed using fMRIPrep (version stable 20.0.2,^9^), run through NiPype (version 1.8.1,^10^).

### Anatomical data preprocessing

Each T1w (T1-weighted) volume was corrected for INU (intensity non-uniformity) using N4BiasFieldCorrection v2.1.0^11^ and skull-stripped using antsBrainExtraction.sh v2.1.0 (using the OASIS template). Spatial normalization to the ICBM 152 Nonlinear Asymmetrical template version 2009c^12^ was performed through nonlinear registration with the antsRegistration tool of ANTs v2.1.0^13^, using brain-extracted versions of both T1w volume and template. Brain tissue segmentation of cerebrospinal fluid (CSF), white-matter (WM) and gray-matter (GM) was performed on the brain-extracted T1w using fast^14^ (FSL v5.0.9, RRID:SCR_002823). The following templates were selected for spatial normalization: FSL’s MNI ICBM 152 non-linear 6th Generation Asymmetric Average Brain Stereotaxic Registration Model [@mni152nlin6asym, RRID:SCR_002823; TemplateFlow ID: MNI152NLin6Asym], “ICBM 152 Nonlinear Asymmetrical template version 2009c” [@mni152nlin2009casym, RRID:SCR_008796; TemplateFlow ID: MNI152NLin2009cAsym].

### Functional data preprocessing

For each BOLD resting-state run found per subject, the following preprocessing was performed. First, a reference volume and its skull-stripped version were generated using a custom methodology of fMRIPrep.

Next, head-motion parameters with respect to the BOLD reference (transformation matrices, and six corresponding rotation and translation parameters) were estimated before any spatiotemporal filtering using mcflirt [FSL 6.0.3:b862cdd5,^15^]. The BOLD time-series (including slice-timing correction when applied) were resampled onto their original, native space by applying the transforms to correct for headmotion. These resampled BOLD time-series will be referred to as preprocessed BOLD in original space, or just preprocessed BOLD. The BOLD reference was then co-registered to the T1w reference using *mri*_*c*_ or *eg* (FreeSurfer) followed by *flirt* [FSL 6.0.3:b862cdd5,^15^] with the boundary-based registration^16^ cost-function. Co-registration was configured with six degrees of freedom. Several confounding time-series were calculated based on the preprocessed BOLD including framewise displacement (FD), DVARS and three anatomically derived global signals.

FD was computed using two formulations following Power (absolute sum of relative motions,^17^) and Jenkinson (relative root mean square displacement between affines,^18^). FD and DVARS were calculated for each functional run, both using their implementations in *Nipype* [following the definitions by^17^]. The three global signals were extracted within the CSF, the WM, and the whole-brain masks.

Additionally, a set of physiological regressors were extracted to allow for component-based noise correction [CompCor,^19^]. Principal components were estimated after high-pass filtering the preprocessed BOLD time-series (using a discrete cosine filter with 128s cut-off) for the two CompCor variants: temporal (tCompCor) and anatomical (aCompCor).tCompCor components are then calculated from the top 2% variable voxels within the brain mask. For aCompCor, three probabilistic masks (CSF, WM and combined CSF+WM) are generated in anatomical space. The implementation differs from that of Behzadi et al. in that instead of eroding the masks by 2 pixels on BOLD space, the aCompCor masks were a subtracted mask of pixels that likely contain a volume fraction of GM. This mask was obtained by thresholding the corresponding partial volume map at 0.05, and it ensures components were not extracted from voxels containing a minimal fraction of GM. Finally, these masks were resampled into BOLD space and binarized by thresholding at 0.99 (as in the original implementation).

The head-motion estimates calculated in the correction step were also placed within the corresponding confounds file. The confound time series derived from head motion estimates and global signals were expanded with the inclusion of temporal derivatives and quadratic terms for each^20^. Frames that exceeded a threshold of 0.5 mm FD or 1.5 standardised DVARS were annotated as motion outliers. The BOLD time-series were resampled into several standard spaces, correspondingly generating the following spatially-normalized, preprocessed BOLD runs: MNI152NLin6Asym (for realignment to same resolution as atlas), MNI152NLin2009cAsym.

All resamplings were performed with a single interpolation step by composing all the pertinent transformations (i.e. head-motion transform matrices, susceptibility distortion correction when available, and co-registrations to anatomical and output spaces). Gridded (volumetric) resamplings were performed using *antsApplyTran* or *ms* (ANTs), configured with Lanczos interpolation to minimize the smoothing effects of other kernels^13^. Non-gridded (surface) resamplings were performed using *mri*_*v*_*ol2surf*(FreeSurfer). Many internal operations of fMRIPrep use Nilearn 0.9.1 [^21^,RRID:SCR_001362], mostly within the functional processing workflow. For more details of the pipeline, see [the section corresponding to workflows in fMRIPrep’s documentation (https://fmriprep.readthedocs.io/en/latest/workflows.html).

All BOLD signal was denoised by regressing out the following noise parameters 12 measures of head motion and their corresponding derivatives, white-matter and CSF measures and frame-wise displacement and a high and low-pass filter (0.01 Hz & 0.1 Hz) was applied (using customised code generated through Nilearn, version 0.9.1). Note, we do not use CompCor anatomically derived physiological noises as a regressor in this preprocessing.

### Functional MRI Analysis

#### Region of Interest Selection & Functional Connectivity

The denoised functional data underwent BOLD time-series extraction for the 17-network 400-regions of interest (ROI) defined by the Schaefer cortical parcellation^22^. We categorised the regions into the specific cortical networks, based upon the Yeo’s 7-network grouping of the 400 Schaefer atlas parcellation (with an additional network for regions labelled as the tempo-parietal regions from the 17-network parcellation), locus coeruleus^23^ and nucleus basalis of Meynert.^24^ To determine the functional connectivity for each participant, the Pearson’s correlation was calculated for each average BOLD time-series for each unique ROI-to-ROI connection (excluding self-connections for statistical analysis, given that self-connections are correlated as 1).

#### Dynamic Functional Connectivity

Time-varying functional connectivity was calculated for each pair of ROIs across temporal windows. We used the multiplication of temporal derivatives which calculates dynamic functional connectivity for each pair of ROIs.^25^ For each region, *n*, with time points, *t*, a vector of *t-1*, temporal derivatives were calculated and normalized. A matrix of functional coupling between the *ith* and *jth* regions for each time point is multiplied by the temporal derivatives of each pairs of regions across temporal windows (5TRs, 40.66 ms) (code is freely available at https://github.com/macshine/coupling/).


$${\text{MTD}}_{\text{ijt}}=\frac{1}{w}\sum_{t}^{t+w}\frac{\left(dt_{it}\times dt_{jt}\right)}{\left(\sigma_{dt_{i}}\times \sigma_{dt_{j}}\right)}$$

Note that we removed one subject from the time-varying analysis due to the entire time-series being slightly shorter in length – to ease further calculations with graph theoretical metrics that are time-varying in nature.

#### Linear Discriminant Analysis

Linear discriminant analysis (LDA) was used to capture the spatio-temporal dynamics of functional connectivity from the noradrenergic (LC), and cholinergic (nbM) nuclei to rest of the 400 cortical ROIs. Dynamic functional connectivity from the specific seeds mentioned above are then concatenated across subjects and time to generate a 119 × 210 (time point) matrix, by ROIs; in order to reduce instability, we scaled the concatenated data prior to fitting the LDA. Using an eigen-mode LDA (scikit learn), we accounted for imbalances in the number of participants in each group by setting the priors to (0.5:0.5), which forces an ‘equal’ assumption for class specification within the model. Then, we calculated the group-based separation by taking the average LDA coefficients to determine the directionality of the LDA-axis for group-based separation. We back-projected the original functional connectivity as the weights assigned to each region (‘feature’) in the LDA; to determine how the features contribute to the group-separation. From the LDA, we select the top 5% discriminating time-points (largest order of magnitude) across the entire dFC for the corresponding nuclei. We then took the average of those corresponding top-performing time-points from the LDA-separation and look at differences in the overall dFC for the noradrenergic and cholinergic connections across the delirious and non-delirious.

#### Graph Theoretical Measures

In addition, we wanted to quantify whether participants had overall more integrated or segregated network topology. We applied the Louvain algorithm to identify modules of strongly intra-correlated brain regions. Then, to quantify each regions’ integrations across the modules, we calculated the participation coefficient (B_T_) for each time-resolved dynamic functional connectivity matrix (using the Brain Connectivity Toolbox^26^). For a given time-point, T,^***KisT***^ represents the strength of positive connections from region(*i*) to regions within module(*s*), and ^***KisT***^ is the total strength of all positive connections of region(*i*). A participation coefficient near 1 indicates that a region is functionally connected across multiple modules (integration), whereas a value near 0 reflects connections confined largely to its own module (segregation)^27–30^. Module assignments were based on the Yeo’s 17-Networks parcellation.


$$B_{iT}=1-\sum_{s=1}^{n_{M}}{\left(\frac{K_{\text{isT}}}{K_{\text{IT}}}\right)}^{2}$$

To further categorise integrated and segregated network states over time, we applied *k*-means clustering to the region-wise time-resolved participation coefficient data. For this analysis, data from all subjects were concatenated across time, resulting in a matrix of size (subjects × time) × regions, where each row corresponded to the brain state of a region at a specific time point. This approach enabled us to treat each regional time point as an independent observation in clustering space.

*K*-means clustering was implemented using the KMeans algorithm (scikit-learn python 3.12.8). The number of clusters was fixed at *k* = 2, consistent with prior work distinguishing between integrated and segregated states (based of Shine et al., 2016^31^). We used the default parameters, including the k-means++ initialisation method to improve convergence, and ran the algorithm with 100 initialisations (n_init=100) to ensure robust clustering solutions. The algorithm iteratively minimised the within-cluster sum of squares (inertia) to partition the data into two non-overlapping clusters, which were then labelled based on their average participation coefficient values: clusters with higher average values were designated as integrated states, and those with lower values as segregated states (Supplementary Materials, Fig. S1A). This approach allowed us to identify recurring, distinct network states across the entire time series.

#### Phasic Increases in Noradrenergic and Cholinergic BOLD Signal

To identify the relationship between phasic BOLD dynamics of the noradrenergic and cholinergic system in relation to fluctuations in network topology^32,33^, we identified phasic peaks in BOLD signal by identifying time-points in which the original BOLD time-series was greater than or equal to two standard deviations above the mean for the timeseries within a 10TR window; and we excluded any timepoints within the first or last 10TRs of the time-series (avoiding boundary related effects). We then looked at the cross-correlation between the participation coefficient (time-varying) and the proceeding five TRs prior and after peak in BOLD signal for the LC and nbM respectively. We then calculated the statistical difference between the cross-correlation values for the delirious and non-delirious groups, by running a permutation-based independence t-test and determine whether there were significant differences in the cross-correlation between the BOLD dynamics of the LC and nbM in relation to fluctuations in the participation coefficient.

### Statistical Analysis

To determine significant differences in FC between the two groups, we performed a permutation-based independent samples t-test. Group labels were randomly shuffled, and the t-test was conducted for each permutation. The process was repeated for a large number of iterations (e.g., 1,000 permutations) to generate a null distribution, allowing us to identify significant group-wise differences in connectivity at each unique ROI-to-ROI edge. This approach controlled for multiple comparisons across all pairwise connections between cortical regions.

### Logistic Regression

To determine the relationship between multiple different variables we ran a logistic regression to disentangle whether greater segregation relates to delirium-based interactions with other predictive measures.


$$D\sim PC_{i}\times C$$

*D* is the postoperative delirium outcome (binarized outcome, binomial methods), and *PC*_*i*_ is the regional(*i*) participation coefficient and *C* is the predictive measure. We also verified that the preoperative PC values interacted with the difference in postoperative EEG slow-wave activity (Postoperative – Preoperative Oz-delta power) in their association with postoperative delirium. For appropriate model selection, we verified the best performing model by using Akaike Information Criterion (AIC) comparison for 3 versions of the model including predicting delirium outcome from just PC (AIC = 140), difference in postoperative EEG slow-wave activity (AIC = 77.7), PC interacting with difference in EEG slow-wave activity (AIC= 65.56), and for PC covariates with difference in EEG slow-wave activity (AIC = 79.66) (Supplementary Materials, Fig. 2A).

In addition, we ran supplementary analysis predicting delirium outcome from average FC for LC and nbM, as well as their relationship with interaction with difference in slow-wave EEG activity (Supplementary Materials, Fig.S3A-D).

Lastly, we conducted a preliminary exploratory analysis to disentangle the relationship between cholinergic functional connectivity and blood-biomarkers that are indicative of neuropathological insult. We ran Pearson’s correlation between the average nbM FC for each subject compared to the blood-biomarker phosphorylated Tau Protein (pTau-181)^34^, we confirmed the linear relationship significance with FDR corrected p-values (Supplementary Materials, Fig. 9).

### Gene-expression Analysis

To test the relationship between cholinergic gene expression data obtained from the Allen Human Brain Atlas^35,36^ (AHBA); we preselected expression profiles relating to the cholinergic system (18 relevant genes) including nicotinic receptor (nAChRs) subunits (CHRNA1, CHRNA2, CHRNA3, CHRNA4, CHRNA5, CHRNA6, CHRNA7, CHRNB1, CHRNB2, CHRNB3, CHRNB4), muscarinic receptors (CHRM1, CHRM2, CHRM3, CHRM4, CHRM5), cholinergic synthesis (CHAT), vesicular transport (SLC18A3, SLC44A2, SLC44A5, SLC5A7, SLC44A1) and cholinergic degradation enzymes (ACHE, BCHE). All data was preprocessed^37–39^ and microarray probes were filtered to retain only expression levels above background threshold of more than 50%. For each gene, the probe with highest differential stability across donors were selected as representative. Then, gene expression values were aggregated and normalised across the cortex using an outlier-robust, scaled sigmoid transformation. The gene data were registered to standard MNI 2mm space, to enable mapping between the AHBA gene expression and our 400 cortical Schaefer parcellation. Expression profiles of our preselected genes were extracted for each region and z-scored. We performed a principal component analysis (PCA) to capture a whole brain low-dimensional resolution of cholinergic gene expression and selected the top principal components that explained 80% variance in the data (Supplementary Materials, Fig. S5). We then ran Pearson correlation across the average participation coefficient for each group (delirium and non-delirious); and combined both groups together and the top 8 principal components, with spin permutation testing (1000 iterations) to deal with spatial autocorrelation and FDR correction (all correlations plotted Supplementary Materials, Fig. 6 & 7). We also performed validation of the best performing principal component in relation to computing the lowest FDR p-value, strongest absolute correlation (ranking the principal components in accordance) and calculated the effect-size (determined small, medium and large based upon Cohen’s D guidelines) (Supplementary Materials, Fig. S5B). In addition, using the Williams-Hotelling formula, we compared the correlation coefficients between the principal components (with shared common variable the average participation coefficient), to determine whether the principal component correlations with average participation coefficient are significantly distinct. We performed 28 pairwise comparison (comparing each principal component correlation with each other); 14 remained significant after FDR correction. For instance, the second principal component had the largest William’s T-Test *T*=+8.98, p=0.001 (FDR) (when compared against principal component 1) (Supplementary Materials, Fig. S5E-F). We also ran correlation analysis across selected genes that contributed to the top-performing principal components and strongest rho correlation (number 2). We also ran the above analysis with the average functional connectivity from the nbM to the cortex (Supplementary Materials, Fig. S8).

## Supplementary Material

Supplementary Files

This is a list of supplementary files associated with this preprint. Click to download.

- SupplementaryMaterials.docx

Supplementary materials are available online.
